# Model for Electrical Field Distribution in the Human Esophagus during Stimulation with Patch and Ring Electrodes

**DOI:** 10.1155/2011/562592

**Published:** 2011-11-15

**Authors:** Christina Brock, Romulus E. Lontis, Flemming H. Lundager, Peter Kunwald, Asbjørn M. Drewes, Hans Gregersen

**Affiliations:** ^1^Mech-Sense, Department of Gastroenterology, Aalborg Hospital, Aarhus University Hospital, Mølleparkvej 4, 9000 Aalborg, Denmark; ^2^Department of Health Science and Technology, Aalborg University, 9220 Aalborg, Denmark; ^3^Sino-Danish Center for Education and Research, 8000 Aarhus, Denmark

## Abstract

*Introduction*. Electrical stimulation is used in experimental human pain models. The aim was to develop a model that visualizes the distribution of electrical field in the esophagus close to ring and patch electrodes mounted on an esophageal catheter and to explain the obtained sensory responses. *Methods*. Electrical field distribution in esophageal layers (mucosa, muscle layers, and surrounding tissue) was computed using a finite element model based on a 3D model. Each layer was assigned different electrical properties. An electrical field exceeding 20 V/m was considered to activate the esophageal afferents. *Results*. The model output showed homogeneous and symmetrical field surrounding ring electrodes compared to a saddle-shaped field around patch electrodes. Increasing interelectrode distance enlarged the electrical field in muscle layer. *Conclusion*. Ring electrodes with 10 mm interelectrode distance seem optimal for future catheter designs. Though the model needs further validation, the results seem useful for electrode designs and understanding of electrical stimulation patterns.

## 1. Introduction

The regulation of esophageal function through extrinsic and intrinsic nerves is complex [1, 2]. Transmucosal electrical stimulation of esophagus with ring and patch electrodes has been widely used in visceral experimental pain models [3, 4]. Electrical stimulation is nonphysiologic and depolarizes all free nerve endings bypassing nociceptive-specific receptors. The wide dynamic range from sensation to pain threshold as well as the exact control of the stimulus onset emphasizes the importance of exploring basic sensory mechanisms as well as the effect of analgesics. 

Controversies exist regarding design and type of electrodes for esophageal electrical stimulation, that is both ring electrodes and patch electrodes are commonly used [5–11]. Patch electrodes glued to a distending bag will, depending on the bagfilling, squeeze against the mucosa. On the other hand, ring electrodes mounted on the catheter may stimulate only a limited part of the mucosa due to the varying contact with the mucosa as the cross-section of the lumen is not circular [12].

We hypothesized that a three-compartment 3D finite element model (FEM) could compute the electrical field in the mucosal and muscle layers and in the serosa and tissue surrounding the esophagus (named “lung” in the text) during electrical stimulation. The proposed model included analysis of currents induced by both types of electrodes and with different contact surfaces for the ring electrodes. The analysis of the stimulation volume relates to the distribution of the electric field and the nerve density in each layer. The bigger the stimulation volume, the higher the number of nerve endings contributing to the response. 

Data from a human pain experiment was used in the model developed in this study to obtain information of the sensation related to stimulation volume.

The aims of the study were to develop the FEM model of the electrode/tissue geometry and provide results of the electrical field distribution in relation to sensation levels reported.

## 2. Material and Methods

### 2.1. Finite Element Model

FEM provides analysis of the electric field in a defined volume conductor. In the developed model the conductor represents two anatomical layers of the esophagus. The geometry of the esophagus was determined in an earlier experiment by use of endoscopic ultrasound images [13]. Hence, the thicknesses of the mucosa and muscle layers used in this study were 0.85 and 2.72 mm resulting in a total wall thickness of 3.57 mm. The size of the mesh was chosen such that at least five mesh points were defined for a uniform domain, that is, at least five points on each side of the interface between the two domains. For simplicity and as a first approach, the model assumed isotropic conditions. The conductivity for the mucosa layer was set to 0.05 S/m according to the literature specifications [14] and for the muscle layer to 0.53 S/m [14]. The volume surrounding the two layers was considered infinite with a conductivity of 0.27 S/m [14] consisting of serosa and lung tissue. The conductivity values are valid for low-range frequencies (<100 kHz). A three-compartment FEM was established which enabled analysis and comparison of the electrical field distribution for bipolar patch electrodes glued to a short bag for distension and bipolar ring electrodes mounted on the catheter with 2 mm, 10 mm, and 20 mm interelectrode distances, respectively. The model visualized the electrical field distribution with different electrode designs. The mucosa surface geometry (round or elliptical) was taken into consideration in the computations related to the ring electrodes.

The function of the volume conductor was

(1) $$\begin{array}{c} I_{\text{response}}=\;f\left({Vol}_{E},\;{\Delta t}_{\text{stimulus}}\right) \end{array}.$$

The FEM was based on the differential equation for the current conservation law:

(2) $$\begin{array}{c} \nabla \cdot J=-\frac{\partial Q}{\partial t} \end{array},$$

in which **J** was the current density in the volume conductor and **Q** was the electric charge density injected at the electrode. The current density was expressed as a function of conductivity (**σ**) of the volume conductor and thus the intensity of the electric field (**E**) and the electric potential (**V**) according to Ohm's law (differential) as follows

(3) $$\begin{array}{c} J=\sigma E=-\sigma \nabla V \end{array}.$$

The equation used for the FEM was of

(4) $$\begin{array}{c} \nabla \cdot \left(\sigma \nabla V\right)=\frac{\partial Q}{\partial t} \end{array}.$$

The output of the model was used to visualize the 3D electrical field distribution. Furthermore, the electrical field in which the nerves could be activated was calculated. The threshold was set to 20 V/m [15].

### 2.2. Human Experiments

**Figure 1:**
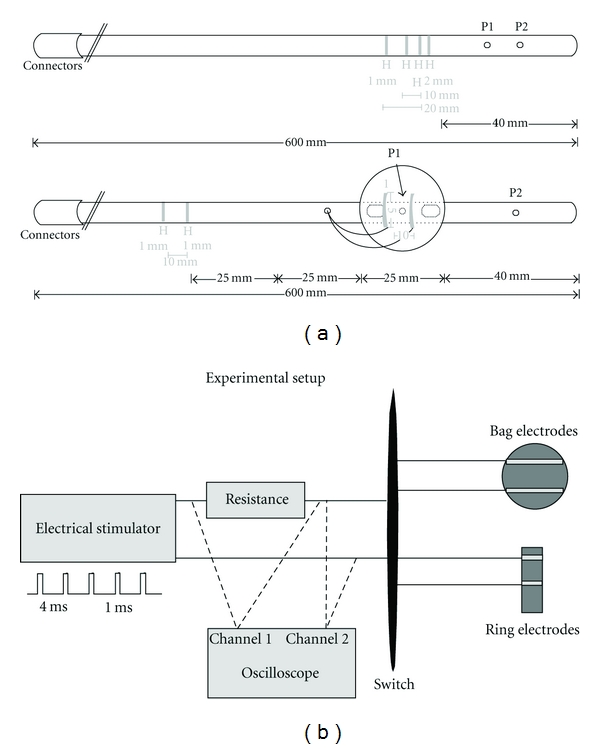
(a) Catheter was developed and designed to compare electrical stimulation with ring electrodes placed on the catheter using different electrode distances. Another catheter was designed for comparison of electrical stimulation with ring electrodes placed on the catheter and patch electrodes placed on a small inflatable bag. (b) The experimental setup. The potential across a known resistance was measured as U1 to verify the output of the electrical stimulator. The verified current intensity was used to calculate the transmural impedance of the total esophageal wall from the measured potential U2. P1 and P2 are pressure recording sites on the probe.

Two flexible esophageal catheters were constructed (Ditens A/S, Hornslet, Denmark). One catheter had four stainless steel electrodes forming three sets of electrodes placed on the catheter with electrode distances of 2, 10, and 20 mm (Figure 1(a)). Transmural impedance and sensory scores (see below) related to the different designs were obtained. Based on these findings another catheter was designed with one pair of ring electrodes and one pair of patch electrodes placed on the exterior of an inflatable nonconductive polyurethane bag of 6 mm in length. The fully inflated bag contained 6 mL saline, and the maximum un-stretched diameter and circumference were 28 and 88 mm (Figure 1(a)). The distance between the two ring electrodes and the two patch electrodes was 10 mm. In the catheter containing the patch and ring electrodes, two lumens were used for inflating and deflating the bag. Two other lumens were used for pressure measurements, one in the bag and one proximal to it, to ensure correct position of the catheter in the esophagus. The surface area of the patch and ring electrodes was equal.

### 2.3. Study Protocol

The in vivo data were obtained from two healthy male volunteers, who neither suffered from any upper gastrointestinal symptoms nor received any medication. The subjects had earlier undergone esophageal endoscopy, which excluded pathologies such as esophagitis and hiatus hernia. The local Ethical Committee approved the study protocol (VN 2003/120 mch) conforming to the Declaration of Helsinki. Oral and written informed consent was obtained. The volunteers were paid 150 DKK/hour to participate.

Prior to the study, the subjects were instructed to use the 0–10 electronic visual analogue scale (VAS) where 0: no perception; 1: vague perception of mild sensation (sensation threshold (ST)); 2: definite perception of mild sensation; 3: vague perception of moderate sensation; 4: definite perception of moderate perception. For *painful* sensations the subjects used the scale from 5–10 anchored at 5: pain detection threshold (PDT); 6: slight pain; 7: moderate pain; 8: medium pain intensity; 9: intense pain, and 10: unbearable pain. The scale has been described in detail previously [16] and has proven to be robust and reliable during esophageal stimulation [9]. Stimulations were repeated three times, and the average intensity was calculated. For safety reasons an ECG was obtained throughout the experiment. The current intensity was increased in steps of 1 mA until the PDT was reached. Intermittent sham stimuli with the same or lower current intensities were randomly delivered to blind the subjects for a predictable stimulation pattern. 

Initially, the catheter with the three pairs of ring electrodes was placed in the esophagus with the excitation electrode positioned 5 cm above the lower esophageal sphincter. Series of three stimulations for each interelectrode distance were done until PDT, beginning with 2 mm and ending with 20 mm.

After withdrawal of the first catheter, the other catheter, with both patch and ring electrodes was positioned so that the center of the bag would be placed 5 cm proximal to the lower esophageal sphincter, and stimulation was done with the bag inflated to volumes of 2, 4, and 5 mL (the latter was associated with vague perception). Stimulation with higher volumes was not done in order to avoid mechanical stimulation of the esophagus. 

The electrodes regardless of the design were connected to a switchbox with a known electrical resistance and a two-channel oscilloscope (HP 54615B, Hewlett-Packard, Geneva, Switzerland). To validate good mucosal contact, the oscilloscope measured the transmural electrical potential (Figure 1(b)). Hence, the impedance of the esophageal wall could be calculated. A computer-controlled constant-current stimulator (IES 230, JNI Biomedical ApS, Klarup, Denmark) applied the electrical stimuli consisting of five rectangular constant-current pulses with duration of 1 ms applied at 200 Hz.

## 3. Results

The electrical field distribution was computed in the esophageal layers. The theoretical impedance provided by the FEM and the experimentally determined impedance are all provided in Table 1. In brief the impedance measured in vivo was higher than that determined by the FEM. Furthermore, the impedance was higher using the ring electrodes than that obtained with the patch electrodes.

**Figure 2:**
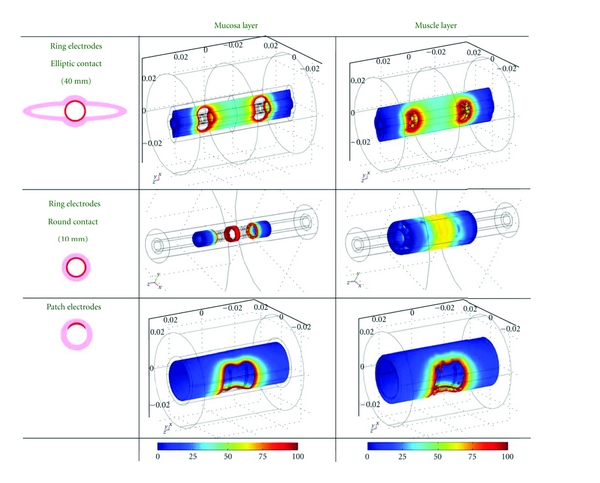
Color-coded graphs of the voltage distribution for the mucosa and the muscle layers computed using FEM. The range of colors from blue to red represents the range from 0 to 100 V/m. The current I was in all cases 20 mA. The first column illustrates the geometry of different contact between electrodes and mucosa. The upper row of graphs is obtained from ring electrodes and an elliptical contact area with the mucosa. The middle graphs are obtained from ring electrodes with a circular contact area to the esophageal mucosa. The bottom graphs are obtained from patch electrodes in contact with the mucosa.

**Figure 3:**
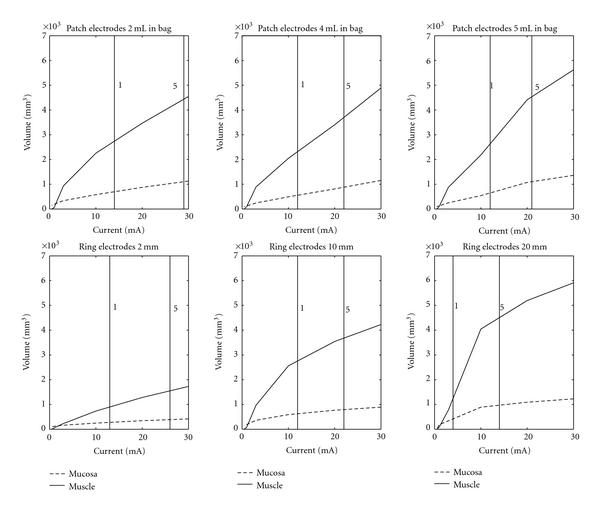
The electrical field which exceeds a threshold of 20 V/m in the three anatomical layers is illustrated. The stimulation current range of the different stimulation electrodes is illustrated as the window between the sensory threshold (VAS 1) and the pain detection threshold (VAS 5).

The model demonstrated a more homogeneous and symmetrical pattern around the ring electrodes regardless of the surface contact compared to a saddle-shaped pattern around the patch electrodes (Figure 2). Increasing the distance between the ring electrodes increased the stimulation volume both at ST and PDT especially in the muscle layer (Table 1). In the example given, the computed electrical field at PDT in the mucosa increased from 388 to 971 mm^3^ when the electrode distance increased from 2 to 20 mm whereas the electrical field in the muscle layer was three fold larger ranging from 1551 to 4504 mm^3^ for the 2 and 20 mm electrode distances (Table 1 and Figure 3). Inflating the bag with the patch electrodes only marginally changed the electrical field in both the mucosa and muscle layer (Figure 3). The stimulation current range expressed as the stimulus intensity between ST and PDT was 13, 14, and 10 mA when stimulating with the three different electrode distances and 15, 10, and 9 mA when stimulating with the patch electrodes on a bag with different filling.

## 4. Discussion

The electrical field distribution in esophagus was computed using a three-compartment 3D FEM assigning different electrical properties to each esophageal layer. The model was used to explain the sensory response to electrical stimulation of the human esophagus using electrode designs commonly used in pain laboratories. Both experimental designs were easy to use, and previous data have shown high reproducibility [9, 17]. The ring electrode design with 10 mm interelectrode distance provided the best stimulation current range and the most homogenous distribution of the electrical field and is the most flexible solution that can be built in to different catheter designs.

## 5. Methodological Considerations

The model was constructed to obtain information of the electrical field distribution in the mucosa and muscle layers of the human esophagus. Electrical stimulation depolarizes the nerve afferents and generates an action potential, which among other factors depends on the direction of the electrical field [18]. The mucosa layer consists of both muscle cells and the mucous membrane. Individually they have different conductivity, 0.5 S/m for muscle and 0.001 S/m for mucosa. Globally the whole layer can be modeled with an equivalent conductivity, for example, between 0.01 S/m and 0.2 S/m. Thus the value of 0.05 S/m was chosen. Based on the dielectric properties of the tissues, the conductivity of the second and third layer was set to 0.53 S/m and 0.27 S/m corresponding to the muscle layer and the surrounding serosa and “lung.” 

An electrical field exceeding 20 V/m in each of the two esophageal layers was considered to activate the sensory afferents. The threshold of 20 V/m [15] corresponds to the threshold for action potential in a nonspecific nerve. Thus, the volume distribution resembles the anatomical layers in which the nerves can be recruited. To visualize the electrical field, it was computed at alternative thresholds. For example a threshold of 100 V/m reflected decreased involvement of the muscle layer whereas a threshold of 3 V/m resulted in increased involvement of the muscle layer. Using the subjective sensory scores as in the FEM, new information was provided regarding the field and involvement of the anatomical layers of the esophagus. 

Different aspects must be considered for development of catheters containing electrodes. Ring electrodes have the advantage that they can be placed almost anywhere on any catheter. On the contrary patch electrodes can be placed on bags, but mounting on the outside of a bag prevents nasal intubation. Electrical contact with the mucosa may be improved by pressure exerted by the bag. However, in the current study inflation of the bag diminished the stimulus intensity, which likely reflects the combination of electrical and mechanical stimulation. In previous experiments atrial capturing was observed during electrical stimulation of the esophagus [19]. Knowledge of the position of the patch electrodes gives the investigator the opportunity to rotate the catheter with the electrodes in order to minimize heart stimulation [16]. On the other hand atrial captures are not of clinical importance, and we never observed arrhythmias in our lab. Hence, it is considered a safe procedure irrespective of electrode design.

## 6. Electric Field

The model demonstrated a more homogeneous and symmetrical electrical distribution around the ring electrodes compared to the saddle-shaped pattern around the patch electrodes. We used the sensory and pain detection thresholds from the obtained experimental data to compare between the simulated and in vivo response. Increased distance between the ring electrodes increased the electrical field which was most prominent in the muscle layer. Furthermore, it lowered the stimulus intensity at both the sensory and pain detection thresholds. 

The sensory nerve endings in the mucosa played only a minor role in the sensory and pain experience (Figure 3). Animal studies have documented that distension-sensitive afferent responses from the muscle layers devoid of the underlying mucosa-submucosa and the serosal membrane are similar to those obtained in the intact wall [20]. The current findings support this. Even though the obtained sensory scores contributed only with unspecific information of the afferent pathways, the nerve endings in the muscle layer contributed most to the pain processing (Figure 3). 

A major factor influencing the impedance is the area of the electrodes that is in contact with the mucosa. Ideally the mucosa will be in contact with the whole electrode surface. Distending the small bag with patch electrodes only marginally changed the electrical field distribution in the mucosal and muscle layers, most likely because the electrodes are squeezed against the esophageal wall protruding the longitudinal folds. The experimental data proved good mucosal contact, even at small volumes of the bag. However, it will be difficult to rule out whether patch electrodes result in altered sensation due to possible mechanical stimulation by the bag position. On contrary it is expected that the mucosal contact to the ring electrodes will vary more, ranging from being round (radius of 1.5 mm) to elliptical (the two semi axes 0.7 and 7.5 mm), depending on the catheter location in the esophageal lumen. However, the concerns regarding the insufficient mucosal contact using the ring electrodes, especially in the elliptical surface pattern, could not be verified experimentally in the current setup.

## 7. The Electrical Stimulus

The nerve excitability in motor axons is followed by a refractory period of approximately 3 milliseconds [21] in which the hyperpolarized membrane either requires a larger potential to activate relative or is unable to be activated absolute. A single pulse for electrical stimulation could have been advantageous as it allows better separation between ST and PDT [22]. However, we used a train consisting of five constant-current pulses, each with duration of 1 ms applied at 200 Hz, as it has proven to be robust and reproducible [9, 17, 23]. The stimulus will roughly excite the nerve endings within the field five times [24], even though the subject will experience the five-pulse train as one stimulus. The pain perception is a summation of the individual excitation from the contributing sensory afferents. However, because the nerve density in the different esophageal layers is unknown, we can only predict an association between the stimulation volume and the subjective pain score. Impedance in the human esophagus can vary due to different local conditions in the esophageal mucosa and saliva production [25], but also food or refluxed gastroduodenal content [26] can explain that we measured higher impedance in vivo than in the simulations. However, a difference in this parameter may also occur due to limitations of isotropic models.

It is desirable to obtain a large stimulation current range using ST and PDT as end points. This was obtained from the ring electrodes with 2 mm (stimulation current range 13 mA) and 10 mm (stimulation current range 14 mA) electrode distance and for patch electrodes on the bag with 2 mL filling (stimulation current range 15 mA). The stimulation current range at 20 mm electrode distance was 40% decreased compared to lower interelectrode distances. Thus the electrode pair should have no more than 10 mm interelectrode distance. On the other hand, the electric field in the esophagus, created by the use of rings with only 2 mm in distance, is less sensitive partly because the esophageal nerve density is unknown and likely unequally distributed. Therefore the inter-electrode distance of 10 mm is preferable. 

## 8. Conclusions

We describe a finite element model of the electrode/tissue geometry and give the results of experimental measurements. Ring electrodes placed with an electrode distance of 10 mm provided the best stimulation current range and the most homogenous field and seem the most flexible solution for catheter designs. Though further development to an anisotropic mode and more validation are needed in future studies, it seems a fair recommendation to use this design in pain experiments using electrical stimulation of the esophagus.

## Figures and Tables

**Table 1:** For simplicity and as a first approach, the model assumed isotropic conditions.

Impedance and electrical field volume	Ring	Ring	Ring	Patch	Patch	Patch
Electrode design						
Interelectrode distance [mm]/volume (mL)	2/−	10/−	20/−	10/2	10/4	10/5
Experimental Impedance (kΩ)	2.41 ± 0.12	2.89 ± 0.25	2.95 ± 0.25	1.36 ± 26	1.46 ± 0.31	1.11 ± 0.05
Computed Impedance (kΩ)	0.94 ± 0.01	1.05 ± 0.01	1.08 ± 0.01	0.86 ± 0.01	0.81 ± 0.01	0.79 ± 0.01
Sensory threshold (mA)	13	8	4	14	12	12
Pain detection threshold (mA)	26	22	14	29	22	21

Computed electrical field volume for the mucosa						
At the sensory threshold (mm^3^)	276	626	419	697	557	647
At the pain detection threshold (mm^3^)	388	793	971	1102	880	1104

Computed electrical field volume for the muscle						
At the sensory threshold (mm^3^)	898	2759	1249	2739	2318	2631
At the pain detection threshold (mm^3^)	1551	3681	4504	4437	3701	4536
